# Peripheral intravenous catheters: the impact of compliance with standards on patient-reported experience measures

**DOI:** 10.1177/0310057X251387731

**Published:** 2025-12-02

**Authors:** Bhavna Brijball, Renata Hadzic, Alisa Shvartsbart, Bernadette R Findlay, Matthew Harland, Timothy De Solom, Fatemeh Emadi, Jonathan Penm, Jennifer A Stevens

**Affiliations:** 1 School of Medicine, University of Notre Dame, Sydney, Australia; 2 Department of Anaesthesia, St Vincent’s Hospital, Sydney, Australia; 3 School of Clinical Medicine, UNSW, Sydney, Australia; 4 School of Pharmacy, University of Sydney, Sydney, Australia; 5 Department of Pharmacy, Prince of Wales Hospital, Randwick, Australia

Although peripheral intravenous catheter (PIVC) insertion is a frequent and often essential part of inpatient treatment, a significant proportion of patients do not receive recommended PIVC care. A study across 51 countries comparing practices with recommendations found widespread variation. Peripheral intravenous catheters were inserted at inappropriate sites (69%), covered with substandard dressings (20%), showed visible signs of redness and swelling at the insertion site (10%), and malfunctioned (e.g. leaked) (10%).^1^ Poorly placed and secured PIVCs have been associated with worse patient-reported outcome measures including pain, distress, and sleep disturbance from alarming, occluded pumps.^2,3^

Australian standards include four observable criteria regarding PIVC placement and securement: placement in an area of non-flexion, use of extension tubing, securement to minimise movement, and use of an appropriate dressing. They also highlight the importance of collaborating with consumers to monitor their experience and facilitate better outcomes.^4^

The primary objective of this study was to explore the relationship between compliance with national standards and PIVC-related sleep disturbance. The secondary objective was to expand the limited research that exists on consumer perspectives of PIVC placement and care via thematic analysis of patients’ comments on how their PIVC experience can be improved.

This study was conducted at St Vincent’s Hospital, Sydney. Ethics approval was granted by the St Vincent’s Hospital Human Research Ethics Committee (2023/ETH00066). Participants were inpatients aged 18 years or older who had a PIVC in situ for at least two nights, were not in the intensive care unit, did not have a documented cognitive impairment, and spoke English.

Compliance with observable criteria of the national standards was audited by visual inspection and rated from zero (no criteria met) to four (all four criteria met). Participants rated disturbance to their sleep from their PIVC and pump alarm noise from zero (no disturbance) to four (a lot of disturbance) for a total score of zero to eight. Patients were given the opportunity to comment on one thing that would improve their PIVC experience.

Compliance was calculated as the percentage of PIVCs fulfilling one, two, three, or all four observable criteria of the standards. A Spearman’s rank-order correlation was used to evaluate the relationship between the number of criteria met and sleep disturbance. It was hypothesised that more optimally positioned and secured PIVCs would correlate with less sleep disturbance. A power analysis indicated a sample size of 138 to detect a correlation coefficient of 0.3 or more, at 95% power and a two-sided significance level of 5%. Thematic analysis was carried out using Braun and Clarke’s six-step process, whereby two authors (FE and JP) independently completed the first three steps, then worked together in the fourth step to generate key themes.

A total of 139 participants (107 men) with a mean age of 62.1 years (standard deviation (SD) of 18.6) had their PIVCs audited and reported on sleep disturbance. The use of an appropriate dressing was high (94.2%). Securement to minimise movement was also sound (70.1%). Use of extension tubing (52.5%) and placement in an area of non-flexion (48.2%) was poor. Compliance with three or all four criteria was observed in 34.5% and 24.5% of PIVCs, respectively.

Participants rated their sleep disturbance as a mean of 2.07 (SD 2.16). A significant correlation was found between the number of criteria met and sleep disturbance, such that greater compliance was associated with less sleep disturbance (*r_s_* = −0.049, *P* = 0.023).

A subset of 87 patients (63%) provided responses on how their PIVC experience could be improved. See Table 1 for examples of patient comments.

**Table 1.** Themes and sample quotes.

Theme 1: Discomfort and inconvenience from PIVC placement site
‘Not in the cubital fossa as it is tender and makes the pump alarm.’ ‘Don’t put it in my elbow crease because it makes it harder to eat and sleep.’ ‘In the inside elbow crease, every time I moved my arm a little bit it set off the machine, which was a nightmare day and night.’ ‘I can’t eat because every time I bend my arm it bends my cannula, which wrecks it and I have to have another one put in. I try to eat with my left hand.’
Theme 2: Preference for alternative site of PIVC placement
‘Put cannula lower than the crease of my arm so I can bend my arm.’ ‘Put them in the forearm rather than elbow to minimise the possibility of the pump making noise.’ ‘Put them in the forearm rather than elbow as this makes it difficult to move around during the day.’
Theme 3: Impact of alarms and beeping
‘The beeping of the alarm is like water torture. They should be using it against Guantanamo terrorists.’ ‘The pump alarms every time I bend my arm.’ ‘Beeping keeps me awake.’ ‘I had a cannula in the elbow crease of my right arm and I am right-handed so the alarm kept going off.’
Theme 4: Securing PIVCs
‘Make it more secure. I can’t move without it pulling, and it started to bleed at the skin.’ ‘Please wrap it properly and make sure it doesn’t get caught on stuff.’ ‘The cannula catches on bedsheets and causes pain.’ ‘I like the little extension part because I can move my arm more without pulling the cannula out.’
Theme 5: Documentation and monitoring
‘Consistently evaluate it and secure it. If the care and management is there, they stay for longer.’ ‘Make sure the date is on the dressing so you can see how long it’s been in.’

PIVC, peripheral intravenous catheter.

Five themes were identified, as described in the following.

## Discomfort and inconvenience related to PIVC placement site

Participants reported that placement in the cubital fossa, as a high-movement area, resulted in tenderness and pain, and limited performance of daily tasks including eating, dressing, and self-care. Participants also reported disrupted sleep as they struggled to position their arm comfortably. Pump alarms triggered by bending the arm further exacerbated discomfort and prevented proper rest.

## Preference for alternative site of PIVC placement

Participants expressed a preference for placement in areas less affected by movement. The forearm, especially the distal anterior forearm, was less painful, allowed for greater movement, and reduced interference with daily activities. Many participants also noted that a more visible location, such as the forearm or hand, allowed for better monitoring and prevention of accidental pulling or irritation.

## Impact of alarms and beeping

Participants reported frequent frustration and anxiety due to the constant beeping of pump alarms, often triggered by occlusion from placement in high-movement areas. Even minor arm movements triggered alarms, disrupting daily activities and rest, and increased discomfort and stress.

## Securing PIVCs

Participants reported discomfort when PIVCs were not adequately taped or covered as they could become loose or catch on clothing or bedsheets, leading to painful tugging on the skin or even re-insertion.

## Documentation and monitoring

Participants highlighted the importance of clear documentation, especially insertion dates on dressings, to track how long the PIVC had been in place. They also called for more frequent changes and monitoring to prevent discomfort and complications from prolonged use.

A 2022 survey of Australian emergency medicine clinicians’ self-reported adherence to the PIVC national standards revealed mixed compliance, with lack of awareness or familiarity identified as one of the biggest barriers.^5^ The results of our study found similarly inconsistent compliance, particularly infrequent use of extension tubing and frequent placement in areas of flexion. Poor compliance had a measurable effect on sleep disturbance in patients, which was further elucidated in comments that stressed the negative impact of placement in the cubital fossa. Routine placement in areas of non-flexion, in line with national standards, would likely decrease discomfort and minimise interference with daily activities. Indeed, Wheeler et al.^2^ reported improved mobility and decreased sleep disturbance due to pump alarms following re-siting from the cubital fossa to the wrist or non-dominant arm. Products such as tape and elasticised tubular bandages have been associated with decreased rates of phlebitis, dislodgement, and occlusion, and may further minimise sleep disturbance from alarms.^6^

As participation was voluntary, self-selection may have favoured individuals with less compliant PIVCs or those with a poorer overall hospital experience. Convenience sampling led to sporadic data collection and may have been impacted by environmental factors such as the time of day and number of other healthcare interactions. The relatively small sample size reflects a metropolitan teaching hospital, such that the results may not generalise to other settings.

For such a common and consequential intervention, PIVC placement and maintenance has a substantial and persistent record of failure. Nearly 70% of participants offered comments on what we could do to improve their experience. These comments showed striking concordance with the national standards, and it was found that higher compliance with standards was correlated with improved sleep. It is clear that further education surrounding best practice is necessary to improve compliance and outcomes.
